# Exploring gender disparities: a survey among orthopedic residents

**DOI:** 10.1186/s10195-025-00847-w

**Published:** 2025-07-03

**Authors:** Rossella Ravaglia, Vittoria Mazzola, Paolo Ferrua, Luca La Verde, Matteo Formica, Pietro Simone Randelli

**Affiliations:** 1 U.O.C. 1° Clinica Ortopedica, ASST G. Pini-CTO, Milan, Italy; 2 Università degli Studi di Milano, Milan, Italy; 3 U.O. Clinica Ortopedica, IRCCS Ospedale Policlinico San Martino, Genoa, Italy; 4 Department of Biomedical Sciences for Health, Università degli Studi di Milano, Milan, Italy; 5 U.O.C. di Ortopedia e Traumatologia, Ospedale San Pietro Fatebenefratelli, Rome, Italy

**Keywords:** Gender discrimination, Orthopedic surgery, Residency, Disparities

## Abstract

**Introduction:**

The representation of women in the medical field has significantly increased in recent decades. However, their presence in surgical specialties, particularly in orthopedic surgery, remains disproportionately low. This study investigates gender discrimination and disparities in Italian orthopedic residency programs, expanding on existing literature, which indicates that female surgeons worldwide face challenges such as fewer promotions, lower salaries, and higher rates of harassment.

**Materials and methods:**

From June to August 2024, the SIAGASCOT Junior Committee conducted a voluntary and anonymous survey among registered male and female orthopedic residents. The survey was distributed via email and social media and included 23 questions covering demographics, training opportunities, perceptions of gender discrimination, and experiences of physical or verbal harassment. Statistical analyses were performed using the Chi-squared test and Mann–Whitney *U* test to compare gender-based differences.

**Results:**

A total of 394 residents were invited to participate in the survey, and 81 residents participated: 46 women (56.8%), 34 men (42%), and 1 respondent who preferred not to disclose his or her gender (response rate: 20.5%). While no significant gender disparities were observed in access to training opportunities, such as international experiences or professional memberships, significant gender differences emerged in perceptions of discrimination. Notably, 84.8% of female respondents reported being considered “unsuitable” for orthopedic surgery solely owing to their gender, compared with 0% of male respondents (*p* < 0.01). In addition, 85% of women reported experiencing verbal or physical harassment, primarily from male superiors or patients.

**Conclusions:**

This study highlights the persistence of gender disparities in orthopedic surgery, with notable differences in perceived discrimination and harassment experiences between male and female residents. Although training opportunities appear to be equally distributed, the reported gender disparities seem to arise from subjective perceptions and cultural attitudes rather than measurable differences. Addressing these disparities requires cultural shifts, mentorship programs, and institutional policies aimed at eliminating harassment and promoting equity, ultimately fostering a more inclusive and supportive environment in orthopedic surgery.

**Level of evidence:**

III.

## Introduction

Over the past few decades, an increasing number of women have entered the medical profession. An analysis of medical school enrollment data in Italy over the last 10 years reveals a steady rise in the number of female students, with a nearly constant growth rate [1]. In addition, after finishing their university studies, the number of female doctors who win a scholarship to work in a hospital is much higher than the number of male doctors [2].

As evidenced by the data from recent years, 67% of residency programs have a higher number of female enrollments (33 programs out of 49). Among these programs, 17 are nonsurgical specialties, 11 are medical service specialties, and only 5 are surgical specialties. These include gynecology, thoracic surgery, general surgery, cardiovascular surgery, and ophthalmic surgery [2].

Despite this trend, the number of women who choose surgical specialties continues to be underrepresented, both in Italy and globally [3]. Between November and December 2020, Women In Surgery Italia (WIS Italia) carried out a comprehensive survey targeting Italian female surgeons across various surgical fields. The findings revealed that a significant majority of these surgeons encounter gender-based discrimination in their professional environment [4].

As highlighted in existing literature, during their training, female students often face, all over the world, more gender discrimination and sexual harassment compared with their male counterparts, especially in general surgery. Once they become surgeons, women tend to experience more gender-based disadvantages such as fewer promotions, lower salaries, and limited opportunities on editorial boards [3].

Analyzing the available data in Italy, it emerges that residencies in orthopedic and trauma surgery are among those with the lowest representation of women, accounting for only 23%, compared with other specialties [1]. This underrepresentation persists also among practicing orthopedic surgeons, as recently demonstrated by Aprato et al. (2025), despite equivalent performance outcomes. [5] Moreover, orthopedic surgery is often perceived as physically demanding and culturally male-dominated, which may discourage female medical graduates from choosing this path.

This study aimed to evaluate the current levels of perceived gender discrimination and harassment among orthopedic residents in Italy, to compare male and female residents’ experiences of harassment, and to investigate any disparities in training opportunities.

## Materials and methods

The scientific society SIAGASCOT, specifically the Junior Committee, distributed a questionnaire to all male and female residents registered with the society. A total of 394 registered resident members of SIAGASCOT Junior were invited to participate via email.

Prior to distributing the questionnaire, data regarding the number of male and female residents in Italy were reviewed; these data were extracted from the Italian Ministry for University and Research (MIUR) website [1].

The invitation included general information about the study, details about the survey, and a consent form, with links to individualized accounts. Participation was voluntary and anonymous, with no compensation provided. The survey was conducted from June to August 2024. A total of three reminder emails were sent at 4-week intervals. The questionnaire was also promoted on social media, including LinkedIn Corporation, Sunnyvale, CA, USA.

The questionnaire was distributed as a Google Form (Google LLC, Mountain View, CA, USA) and consisted of 23 questions. The first three questions were demographic, covering the city of the residency program, the year of residency, and the respondent’s gender. Another three questions addressed training experience, such as international mobility, cadaver labs, and scientific society membership. Subsequently, multiple-choice questions followed: five questions assessed perceived gender-based discrimination, while ten questions focused on experiences of verbal or physical harassment by supervisors, patients, nurses, or colleagues. The five questions on perceived discrimination used a 5-point Likert scale (from “never” to “always”), while the ten questions addressing harassment used a 4-point scale with the options “never,” “once,” “more than once,” and “often.” One question assessed awareness of consensual relationships between residents and senior staff. The final item was an open-ended question for additional comments.

The questionnaire was developed by the SIAGASCOT Junior Committee and internally reviewed by senior members.

Variables with dichotomous responses were described as *n* (%). For ordinal responses on Likert-type scales, the average numerical value corresponding to each answer was calculated. Initially, data were analyzed in aggregate. Subsequently, responses were stratified by gender, and a comparative analysis was performed using the Chi-squared test for categorical variables and the Mann–Whitney *U* test for ordinal variables. All statistical analyses were conducted using Microsoft Excel, with a significance level set at *p* < 0.05.

## Results

According to 2023 data from the Italian Ministry for University and Research (MIUR), female residents constitute 23% of the total number of residents in orthopedic and trauma surgery in Italy.

A total of 81 medical residents responded to the questionnaire, out of 394 invited members (response rate: 20.5%). Among them, 46 were women (56.8%), 34 were men (42%), and 1 respondent preferred not to disclose his or her gender.

Analyzing the data as a whole, the respondents’ distribution by medical school location was relatively balanced across Italy, with a higher concentration in Milan (30), Genoa (10), and Rome (10). Responses were received from 18 orthopedic residency programs across the country.

Participants represented all 5 years of residency, with the following distribution: 1st year—13; 2nd year—21; 3rd year—13; 4th year—18; 5th year—16.

**Fig. 1:**
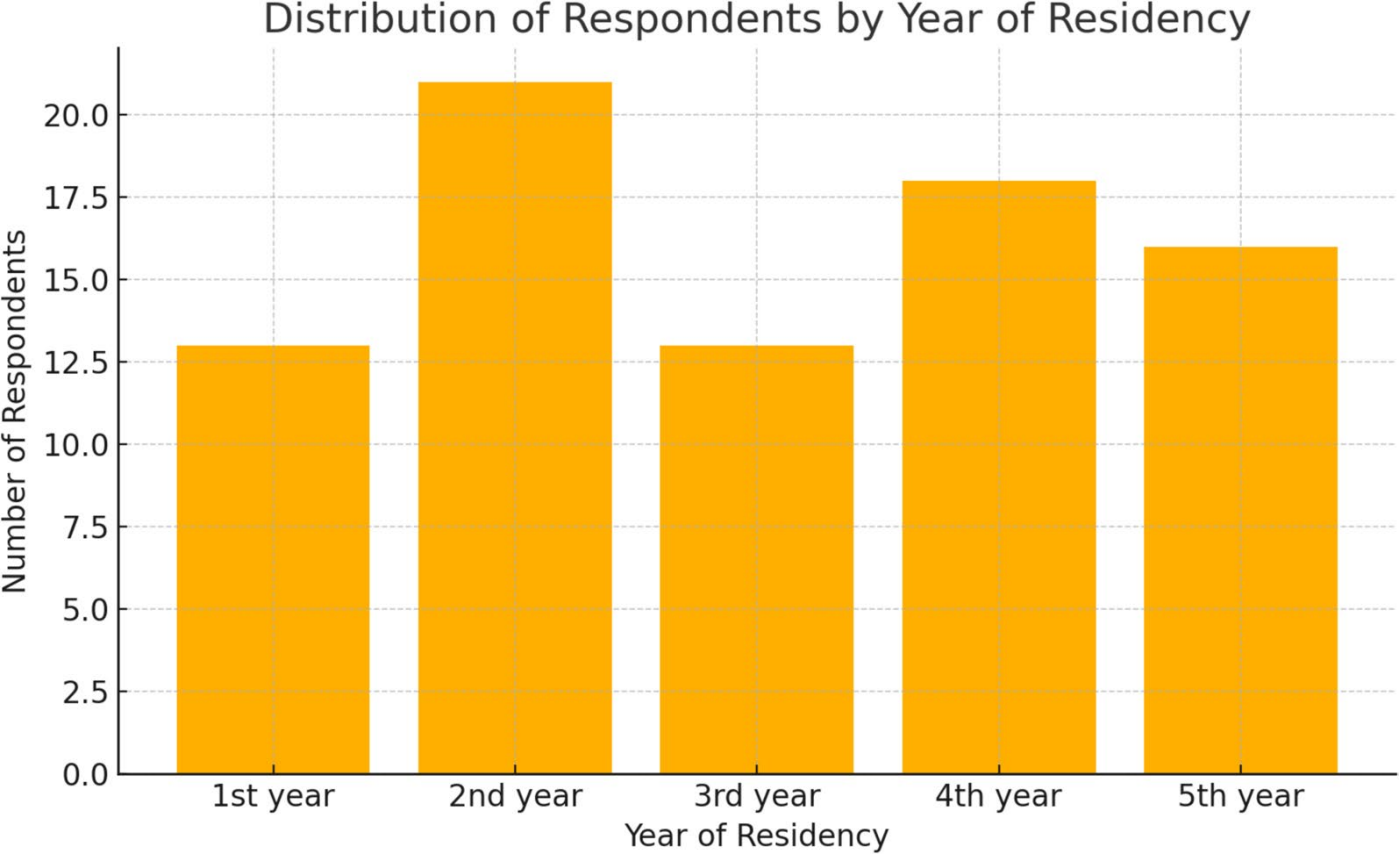
Graph showing the percentage distribution of responses to the question “What year of residency are you in?”

Figure 1 shows the distribution of respondents according to their current year of residency training.

Table 1 presents the responses to the three questions regarding resident training, expressed as *n*(%). No statistically significant differences were found between men and women for any item.

**Table 1:** Responses to the questions regarding resident training, expressed as *n*(%) of “yes”

	Total	Female	Male	*P*-value
Have you had the opportunity to undertake external training periods?	32 (39.5%)	19 (41.3%)	13 (38.2%)	0.78
Are you a member of a scientific society?	61 (75.3%)	35 (76.1%)	26 (76.5%)	0.97
Have you participated in sponsored courses or cadaver labs?	38 (46.9%)	24 (52.2%)	13 (38.2%)	0.31

Table 2 reports the responses to the questions regarding subjective perceptions of gender discrimination. The difference between male and female respondents was statistically significant for all items.

**Table 2:** Responses to questions regarding subjective perceptions of gender discrimination (average ± standard deviation (SD))

		Total	Female	Male	*P*-value
On a scale from 1 to 5, based on your gender, how much do you think you have experienced discrimination:	-From nursing staff	2.10 ± 1.07	2.48 ± 0.98	1.62 ± 0.99	< 0.001
	-From your superiors	2.35 ± 1.17	2.86 ± 0.90	1.55 ± 0.87	< 0.001
	-From patients	2.48 ± 1.36	3.27 ± 0.78	1.15 ± 0.36	< 0.001
	-From colleagues	1.85 ± 0.92	2.24 ± 0.85	1.33 ± 0.74	< 0.001
On a scale from 1 to 5, how much do you think your gender could be an obstacle to career advancement?		2.51 ± 1.39	3.23 ± 0.77	1.19 ± 0.64	< 0.001

In response to the question “Have you ever been told that you were not suitable for orthopedic surgery because of your gender?”, no male resident answered yes, whereas 39 female residents (84.8%) did (*p* < 0.001).

Table 3 presents the responses to questions regarding physical and verbal abuse.

**Table 3:** Responses to questions regarding physical and verbal abuse (average ± SD)

	Total	Female	Male	*P*-value
Have you ever experienced physical harassment from nursing staff of the opposite sex?	1.20 ± 0.56	1.13 ± 0.45	1.29 ± 0.68	*p* = 0.22
Have you ever experienced verbal harassment from nursing staff of the opposite sex?	1.64 ± 0.92	1.74 ± 0.88	1.52 ± 0.97	*p* = 0.14
Have you ever experienced physical harassment from a superior of the opposite sex?	1.27 ± 0.65	1.37 ± 0.74	1.15 ± 0.50	*p* = 0.091
Have you ever experienced verbal harassment from a superior of the opposite sex?	1.68 ± 0.94	2.11 ± 0.99	1.09 ± 0.38	*p* < 0.001
Have you ever experienced physical harassment from a patient of the opposite sex?	1.14 ± 0.44	1.15 ± 0.42	1.12 ± 0.48	*p* = 0.34
Have you ever experienced verbal harassment from a patient of the opposite sex?	1.70 ± 0.91	1.98 ± 0.95	1.33 ± 0.69	*p* = 0.001
Have you ever experienced physical harassment from a colleague (resident) of the opposite sex?	1.09 ± 0.36	1.07 ± 0.33	1.12 ± 0.41	*p* = 0.43
Have you ever experienced verbal harassment from a colleague (resident) of the opposite sex?	1.36 ± 0.80	1.43 ± 0.89	1.27 ± 0.67	*p* = 0.44
Have you ever experienced physical harassment from nursing staff, a patient, a superior, or a colleague of the same sex?	1.15 ± 0.48	1.04 ± 0.21	1.30 ± 0.68	*p* = 0.039
Have you ever experienced verbal harassment from nursing staff, a patient, a superior, or a colleague of the same sex?	1.40 ± 0.84	1.46 ± 0.89	1.33 ± 0.78	*p* = 0.63

As observed from the data presented in Table 3, a statistically significant difference was found between women and men for the questions “Have you ever experienced verbal harassment from a superior of the opposite sex?” and “Have you ever experienced verbal harassment from a patient of the opposite sex?”, with higher scores reported by women.

A statistically significant difference was also found for the question “Have you ever experienced physical harassment from nursing staff, a patient, a superior, or a colleague of the same sex?”, with higher scores reported by men.

Only 23 out of 81 residents responded “never” to all the questions in Table 3—of whom 16 were men (47%) and 7 were women (15%)—a statistically significant finding (*p* = 0.0019). This indicates that 85% of women reported experiencing either physical or verbal harassment.

To the question “Are you aware of consensual relationships between residents and senior staff?”, 70% answered yes.

In response to the final open-ended question, three male residents provided personal comments:

- “An attending flirts with female residents, giving them, among other things, much more attention and opportunities compared with male colleagues.”
- “Female colleagues rarely have their mistakes, even blatant ones, challenged by supervisors. They can say more or less whatever they want, even without appropriate manners.”
- “Male residents are judged based on skill, personality, and flattery, while female residents are judged on skill and appearance, and some take full advantage of this.”

## Discussion

This study explored the widespread gender discrimination in the field of orthopedic surgery in Italy, particularly within residency programs, examining and revealing significant gender differences in both experiences of and perceptions toward gender discrimination among orthopedic surgery residents. One of the most recent studies, conducted by Parini et al. [4], aligns with our findings and addresses specialties such as thoracic, cardiac, and vascular surgery. Their findings indicate that perceived gender bias remains prevalent in these surgical specialties. They noted that discrimination against female surgeons has shifted from overt attitudes to more subtle forms. Many women in surgery continue to report differential treatment based on gender, including receiving less recognition and limited chances for career advancement.

In terms of demographics, responses were collected from residents enrolled in all 5 years of orthopedic training and from 18 different orthopedic residency programs distributed across Italy (Fig. 1).

This geographic and temporal coverage strengthens the representativeness of our sample despite the voluntary nature of participation.

Although gender disparity in orthopedic surgery has been historically underexplored in Italy, recent studies have started to address different facets of the issue. For instance, Aprato et al. showed no differences in clinical outcomes between male and female trauma surgeons [5], while Vitale et al. highlighted the underrepresentation of women in senior authorship positions in high-impact orthopedic journals, indicating persistent academic and structural barriers [6].

A promising finding in our study (Table 1) is that regarding opportunities for overseas training, membership in scientific societies, and participation in courses or cadaver labs, no significant gender differences were observed. This suggests that efforts and interest in the subject are leading to equal opportunities for education and practical training, highlighting the importance of focusing on this issue to drive improvement.

However, it is also clear that there is still a long way to go. In fact, a key finding from our study is that all survey questions regarding subjective perceptions of gender discrimination revealed statistically significant differences between male and female respondents, with women reporting markedly higher levels of perceived discrimination (Table 2).

In our study, gender differences were found to be highly impactful, with 84.8% of women reporting being considered unfit for this type of surgery, compared with 0% of men.

Perhaps the most alarming result of all is that 85% of female residents reported experiencing physical or verbal harassment during residency. Particularly concerning are responses to questions about experiencing verbal harassment from opposite-sex superiors and opposite-sex patients, which were statistically significant, with higher scores for women compared with men, who instead reported higher scores regarding harassment from healthcare professionals or patients of the same sex (Table 3). These findings further confirm the persistence of disparity and discrimination in our hospitals. Similar patterns are reflected in international literature, suggesting that gender bias in orthopedics is a global issue.

As seen in Italy, Koschmeder et al. [7] reported that the percentage of female orthopedic surgeons in the USA increased from 4% in 2016 to 6.5%, and the number of female orthopedic residents rose from 13 to 15%. Although this indicates a positive shift, orthopedics still trails behind other surgical subspecialties in progress. The overall representation of female residents in surgery (including orthopedic surgery, neurosurgery, urology, otolaryngology, plastic surgery, and general surgery) is 33%, significantly higher than the 16% in orthopedic surgery [8].

Marcia Clark et al. also reported that in the USA, orthopedic surgery has the lowest gender representation compared with other medical and surgical specialties, and this trend is evident globally, with very low percentages of women in orthopedics worldwide [9].

Recent Korean studies report results consistent with ours. Choi et al. [3] conducted a survey on gender discrimination that showed a higher perception of discrimination among women, ranging from 20.6 to 87.9%, compared with men.

Studies such as this one and those previously mentioned suggest that merely increasing the number of female surgeons and residents is not sufficient to eliminate gender discrimination. A significant difference in the perception of the issue between male and female surgeons persists. Structured cultural challenges rooted in the history of our society remain to be addressed. An important step, to which studies such as this contribute, is raising awareness of the problem’s existence.

Our findings support the hypothesis that gender disparities in orthopedic surgery are more closely related to cultural perceptions and attitudes rather than performance-based differences.

This is consistent with Aprato et al. [5], who demonstrated no difference in clinical outcomes between male and female trauma surgeons in Italy.

The limitations of this study should be considered when interpreting the results. First, the use of a voluntary questionnaire introduces a risk of self-selection bias, as individuals who have experienced or feel strongly about gender discrimination may be more inclined to participate. Second, the survey relied on self-reported data, which may be subject to recall bias or social desirability bias, potentially affecting the accuracy of the responses. Third, the proportion of female respondents in our sample (56.8%) does not reflect the national average of 23%, as reported by MIUR (2023), and may limit the generalizability of the findings. [1]

Finally, the study was limited to members of SIAGASCOT, potentially restricting the generalizability of the findings to the broader population of orthopedic and trauma surgery residents in Italy.

## Conclusions

This study highlights significant gender disparities in orthopedic surgery residency programs in Italy, particularly in perceived experiences of discrimination and harassment. While no objective differences were found in training opportunities between male and female residents, female participants reported a substantially higher perception of being considered unfit for orthopedics and more frequent episodes of verbal and physical harassment.

A striking 85% of female participants reported at least one instance of gender-based harassment. These findings suggest that although formal training pathways may appear equitable, cultural and interpersonal dynamics still perpetuate gender inequality.

Addressing these challenges requires institutional commitment to fostering a more inclusive culture. Future research should incorporate both objective metrics (e.g., surgical case numbers, academic productivity) and subjective measures (e.g., perception of bias) to better understand and address gender disparities. In addition, the development of structured mentorship programs, anti-harassment policies, and longitudinal monitoring could contribute to a more equitable and supportive environment in orthopedic surgery.

## Data Availability

The datasets generated and/or analyzed during the current study are available from the corresponding author.

## Abbreviations

SIAGASCOT: Italian Society of Knee Surgery, Arthroscopy, Sports, Cartilage, and Orthopaedic Technologies
MIUR: Italian Ministry for University and Research
WIS Italia: Women in Surgery Italia
IRCCS: Institute for Research, Hospitalization and Health Care (Istituto di Ricovero e Cura a Carattere Scientifico)
U.O.C.: Unità Operativa Complessa (Complex Operative Unit)
ASST: Azienda Socio Sanitaria Territoriale (Local Health Service)
